# All-Day Freshwater Harvesting Using Solar Auto-Tracking Assisted Selective Solar Absorption and Radiative Cooling

**DOI:** 10.3390/ma18132967

**Published:** 2025-06-23

**Authors:** Jing Luo, Haining Ji, Runteng Luo, Xiangkai Zheng, Tianjian Xiao

**Affiliations:** School of Physics and Optoelectronics, Xiangtan University, Xiangtan 411105, China202205710216@smail.xtu.edu.cn (R.L.);

**Keywords:** freshwater harvesting, selective solar absorber, radiative cooling, solar tracking

## Abstract

The shortage of freshwater resources has become the core bottleneck of global sustainable development. Traditional freshwater harvesting technologies are restricted by geographical conditions and environmental limitations, making them increasingly difficult to satisfy the growing water demand. In this study, based on the synergistic coupling mechanism of photothermal conversion and radiative cooling, a solar auto-tracking assisted selective solar absorber and radiative cooling all-weather freshwater harvesting device was innovatively developed. The prepared selective solar absorber achieved a high absorptivity of 0.91 in the solar spectrum (0.3–2.5 μm) and maintained a low emissivity of 0.12 in the mid-infrared range (2.5–20 μm), significantly enhancing the photothermal conversion efficiency. The radiative cooling film demonstrated an average cooling effect of 7.62 °C during typical daytime hours (12:00–13:00) and 7.03 °C at night (22:00–23:00), providing a stable low-temperature environment for water vapor condensation. The experimental results showed that the experimental group equipped with the solar auto-tracking system collected 0.79 kg m^−2^ of freshwater in 24 h, representing a 23.4% increase compared to the control group without the solar auto-tracking system. By combining theoretical analysis with experimental validation, this study presents technical and economic advantages for emergency water and island freshwater supply, offering an innovative solution to mitigate the global freshwater crisis.

## 1. Introduction

The scarcity of freshwater resources [1,2], crucial for supporting the sustainable development of human societies and maintaining ecosystem stability, has become a major global issue [3]. Although approximately 70% of the Earth’s surface is covered by water, only 2.5% of the world’s total water resources are freshwater [4]. The World Health Organization predicts that by 2030, half of the global population will experience water shortages, as the demand for freshwater is expected to increase by 40%, exacerbating the imbalance between supply and demand [5].

Self-powered freshwater harvesting devices are a key technological solution to water scarcity [6,7]. Traditional methods, such as rainwater harvesting and groundwater pumping, face significant limitations due to geographic and climatic factors, making it challenging to provide a large-scale and sustainable freshwater supply [8,9]. Seawater, the most abundant water resource on Earth, makes up about 96.53% of the planet’s total water [10,11]. In recent years, seawater distillation technologies powered by electricity or fossil fuels have become widely used [12,13]. However, these technologies face challenges due to their high costs and environmental pollution [14,15]. To overcome the bottlenecks in freshwater harvesting technology, the research team has conducted systematic and innovative research [16]. In solar-driven technology, researchers have improved collector photothermal conversion efficiency using material innovations and structural optimizations [17]. In the research and development of collector materials [18], a breakthrough has been achieved in the preparation technology of selective solar absorbers (SSAs) [19,20]. Prof. Tian and Prof. Xiao et al. [21] successfully prepared a Cu-rich plasma nanostructured SSA with broadband absorption and low mid-infrared emission properties through selective leaching reaction using solution treatment. The SSA achieves over 95% absorptivity in the solar spectrum (0.3–2.5 μm) and less than 5% emissivity in the mid-infrared range (2.5–20 μm), thereby enhancing the efficiency of solar thermal collectors through effective light capture and reduced thermal radiation [22,23,24]. Additionally, researchers have successfully prepared a floating magnetic hydrogel composed of multi-layer graphite nanosheets, polyvinyl alcohol, SiO_2_ aerogel, and Fe_3_O_4_ nanoparticles, providing an innovative solution for solar desalination and water purification with high photothermal conversion efficiency, easy recovery, and sustainability [25]. At the collector structural level, researchers enhanced photothermal conversion efficiency and hydrodynamic performance by designing photonic crystal optical structures, microchannel heat dissipation systems, and optimized flow channel layouts. These results show that the multi-path serpentine photovoltaic-thermal system achieves optimal power output under identical wetted area conditions, with an average total power of 423.84 W m^−2^ [26]. Additionally, sky radiation cooling technology, which relies on the thermal radiation transmission through the atmospheric window, has gained attention due to its passive heat dissipation and zero energy consumption [27,28,29]. Sky radiation cooling relies on heat radiation through the atmospheric window, using radiative cooling coatings with high solar reflectivity and infrared emissivity to achieve efficient thermal dissipation to the cosmic cooling source [30,31,32,33]. During the day, radiative cooling coatings leverage high solar reflectivity to reduce surface temperature, establishing a thermal gradient that drives water vapor condensation. At night, the coatings radiate energy to space via mid-infrared high emissivity, dropping the surface temperature below the dew point to capture atmospheric water vapor for freshwater collection [34,35]. The above technological breakthroughs provide innovative solutions for realizing high-efficiency solar-powered seawater distillation and all-weather freshwater collection.

However, solar collectors are limited to daytime operation due to the periodic changes in the azimuth and altitude of solar radiation, preventing real-time tracking of the sun’s trajectory. For instance, in the northern hemisphere mid-latitudes, the collector incidence angle exceeds 30° outside of noon, causing a photothermal conversion efficiency reduction of over 30% [23]. In addition, salt crystallization from seawater evaporation reduces its evaporation efficiency [36,37]. Sky radiation cooling technology depends on ambient humidity and natural convection. Without active evaporation driving, water collection relies solely on dew condensation, leading to low average daily yields that fail to meet practical needs. Therefore, developing an integrated system combining high-efficiency solar thermal conversion with radiative cooling is crucial to overcoming existing technological bottlenecks [38,39,40].

This paper presents a novel all-weather freshwater harvesting device by integrating interfacial photothermal evaporation, radiative cooling, and solar auto-tracking technologies, with its day-night performance systematically investigated. During the day, the solar tracking device drives the SSA to track the sun in real time, optimizing the incident angle between the photothermal interface and solar radiation to maximize absorption efficiency while minimizing salt deposition. Water vapor from seawater evaporation condenses on the surface formed by the radiative cooling film. At night, the film radiates heat to space, lowering its temperature below the ambient dew point to capture atmospheric moisture. With dual innovations of photothermal-radiative collaboration and dynamic tracking, this device shows great potential in desalination and freshwater collection.

## 2. System Summarization

**Figure 1:**
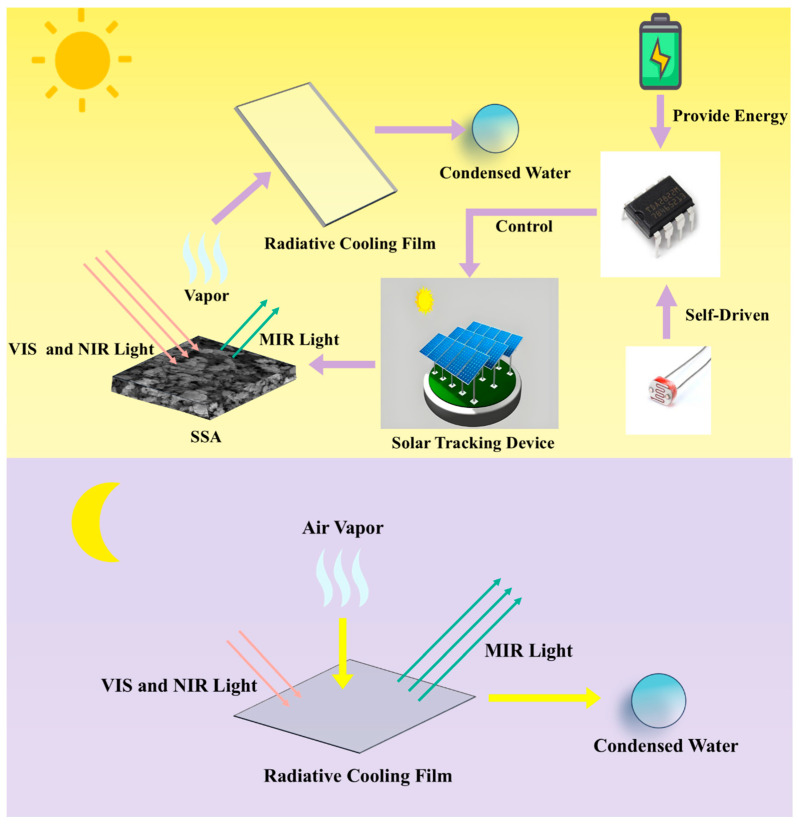
Flow chart of the all-weather freshwater collection device.

The all-weather freshwater collection system designed in this study integrates radiative cooling, solar photothermal conversion, and solar auto-tracking technologies to build an efficient freshwater collection system. Among them, the solar auto-tracking device adjusts the main optical axis of the solar collector in real time, aligning it parallel to the sun’s rays based on feedback from the light sensing mechanism, ensuring maximum light and heat absorption efficiency. In the daytime operation mode, the solar collector device induces seawater evaporation at the interface. The resulting water vapor rises to the surface of the radiative cooling film, where it condenses into liquid water and flows into the collection chamber. At night, the radiative cooling film utilizes its high infrared emission properties to dissipate heat into outer space via long-wave radiation. This process reduces the surface temperature of the film below the ambient dew point, causing atmospheric water vapor to condense and enabling continuous freshwater collection day and night. The workflow of the all-weather freshwater collection device is shown in Figure 1 [10,41].

**Figure 2:**
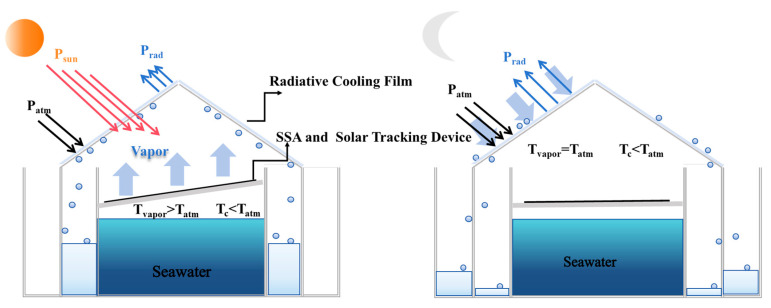
Schematic diagram of the structure of the freshwater collection device and energy flow. P_sun_ is the incident solar power, P_atm_ is the atmospheric radiant power, P_rad_ is the radiant power of the radiatively cooled film, T_atm_ is the atmospheric and ambient temperatures, T_vapor_ is the temperature of the vapors, and T_c_ is the temperature of the polymer coating.

In the integrated all-weather freshwater collection unit, a radiative cooling film is attached to the inclined surface at the top of the device, forming a highly efficient condensation interface. Below it, a suspended SSA module—featuring a hollowed-out design—facilitates upward vapor transport from evaporating seawater. Due to gravity, the condensed water flows along the inclined film and collects in a side tank, enabling continuous freshwater harvesting and storage. The outer tanks are primarily designed to collect fresh water generated on the condensation side, while the inner tanks are dedicated to gathering fresh water produced on the evaporation side. The structure of the freshwater collection device is shown in Figure 2.

### 2.1. Preparation of Solar Collector Module

The solar collector module is fabricated using zinc sheets as the substrate. The substrate pretreatment involves three steps: (1) etching with dilute hydrochloric acid to remove surface oxides and contaminants, (2) sequential ultrasonic cleaning with isopropanol and deionized water to eliminate residual etchant and impurities, and (3) surface drying using compressed air.

Copper nanoparticle deposition was performed using a dip-dry method with a 0.01 mol L^−1^ CuSO_4_ solution prepared by dissolving CuSO_4_·5H_2_O in deionized water under constant magnetic stirring. The solution was then heated to 75 °C in a vacuum drying oven. The pretreated zinc substrates were immersed in the heated copper sulfate solution for 2 min. During this process, a controlled temperature and reaction time facilitated the reduction of copper ions on the zinc surface, resulting in the formation of a uniform copper nanoparticle layer. After deposition, the zinc sheet was rinsed with isopropyl alcohol and deionized water several times to terminate the surface chemical reaction, followed by air drying to complete the SSA sample preparation [42].

### 2.2. Preparation of the Solar Auto-Tracking System

The solar auto-tracking system employs a photoelectric sensing mechanism coupled with a closed-loop control architecture. The core components include the TDA2822 dual-channel power amplifier chip designed by STMicroelectronics (Geneva, Switzerland), geared motors, photoresistors, 10 K resistors, and a power module. Utilizing photoresistors as light sensors, the system continuously monitors variations in solar azimuth and elevation angles. The detected light intensity is converted into voltage signals using a voltage divider circuit. The signals are then amplified and compared differentially using the TDA2822. When the light intensity difference exceeds a predetermined threshold, the control circuit activates the geared motors. The gear transmission mechanism adjusts both the pitch and azimuth angles of the solar collector, maintaining optimal alignment between the SSA’s primary optical axis and incident sunlight. This active tracking mechanism ensures optimal light capture and thermal collection efficiency throughout operational periods. A detailed schematic diagram of the solar auto-tracking system is presented in Figure S1.

### 2.3. Preparation of the Radiative Cooling Module

The radiative cooling module incorporates a composite functional film developed by Ningbo Radi-Cool Advanced Energy Technologies Co., Ltd. (Ningbo, China) [43]. The composite functional film is made of a polymer matrix embedded with inorganic nano-functional fillers, engineered to achieve selective spectral modulation. The film demonstrates high infrared emissivity within the atmospheric window (8–13 μm), enabling efficient heat dissipation to outer space through long-wave radiation. Simultaneously, it exhibits high solar reflectivity across the solar spectrum (0.3–2.5 μm), effectively minimizing solar heat gain. This dual-function spectral selectivity allows the film to achieve remarkable passive cooling performance without external energy input, maintaining a sub-ambient temperature even under direct sunlight. By establishing a stable, low-temperature condensation interface, the film significantly enhances the efficiency of freshwater collection systems.

## 3. Experimental Section

### 3.1. Performance Testing of the Evaporation and Condensation Side

A scanning electron microscope (SEM ZEISS Sigma 300, Oberkochen, Germany) was used to observe the microscopic morphology of the samples. We characterized the SSA’s copper nanostructures (pore size ~50–200 nm), obtaining microstructural parameters including nanoparticle size, spatial arrangement, and surface roughness.

The emissivity spectra of SSA and radiative cooling film in the 2.5–20 μm band were measured using a Fourier transform infrared spectrometer (Nicolet iS50, Thermo Fisher Scientific Inc., Cleveland, OH, USA) at 8 cm^−1^ resolution (0.005 μm wavelength precision), with 128 scans per measurement to enhance the signal-to-noise ratio. Meanwhile, a PerkinElmer Lambda 1050 UV-VIS-NIR spectrophotometer (PerkinElmer, Inc., Shelton, CT, USA) equipped with a 60 mm integrating sphere was used to determine the absorptivity and reflectivity in the 0.3–2.5 μm band. The instrument scanned from 200 to 2500 nm at 200 nm/min with 1 nm resolution and was calibrated against a BaSO_4_ white plate before each test to ensure accuracy. All samples were pretreated in a vacuum drying oven before testing to effectively remove adsorbed water and other volatile impurities from the surface, ensuring that environmental factors did not influence the test results. To reflect the overall optical properties of materials across broad spectral ranges, we employed average optical parameters for analysis. The average absorptivity and average reflectivity were calculated as follows:

(1) $${\bar{\varepsilon }}_{\lambda_{1}-\lambda_{2}}=\frac{\int_{\lambda_{1}}^{\lambda_{2}}\varepsilon (\lambda )B(\lambda ,T)d\lambda }{\int_{\lambda_{1}}^{\lambda_{2}}B(\lambda ,T)d\lambda }$$

(2) $${\bar{\gamma }}_{\lambda_{1}-\lambda_{2}}=\frac{\int_{\lambda_{1}}^{\lambda_{2}}\gamma (\lambda )B(\lambda ,T)d\lambda }{\int_{\lambda_{1}}^{\lambda_{2}}B(\lambda ,T)d\lambda }$$

(3) $$B(\lambda ,T)=\frac{2\pi hc_{0}^{2}\lambda^{-5}}{e^{\frac{hc_{0}}{k\lambda T}-1}}$$

where ${\bar{\varepsilon }}_{\lambda_{1}-\lambda_{2}}$ is the average emissivity, ${\bar{\gamma }}_{\lambda_{1}-\lambda_{2}}$ is the average reflectivity, and B(λ,T) is the irradiance of the blackbody at a wavelength and temperature of T.

A systematic investigation of the radiative cooling film’s condensation performance was conducted using a custom-designed experimental apparatus. The test platform was installed on a school building rooftop to ensure an unobstructed sky view and minimize environmental interference. Temperature measurements were simultaneously recorded for both the film surface and ambient air using high-precision type K thermocouples (±0.1 °C accuracy). Data collection focused on two critical periods: midday (12:00–13:00) during peak solar irradiance and nighttime (22:00–23:00) when ambient temperatures reached their daily minimum. By calculating the temperature difference between the film and the ambient temperature, the condensation efficiency of the radiative cooling film was evaluated under different operating conditions.

### 3.2. All-Weather Freshwater Collection Experiments

The all-weather freshwater harvesting performance experiment was carried out on 1 May 2025, in Xiangtan City, Hunan Province (27.87° N, 112.91° E) under clear sky conditions. During the experimental period, ambient temperatures recorded by the meteorological station ranged from 18 °C to 31 °C. A controlled experimental design was implemented to assess the impact of the solar auto-tracking device on the efficiency of solar thermal collection. The experimental group was equipped with an auto-tracking system based on the TDA2822 chip, ensuring real-time alignment of the SSA’s main optical axis with the sun. The control group, on the other hand, used a traditional fixed collector. All device materials, structural parameters, and environmental conditions were kept consistent between the two groups, with the only variation being the tracking system. During the experimental process, a load cell was utilized to record the amount of freshwater collected.

## 4. Results and Discussion

### 4.1. Evaporation Side Performance Evaluation

**Figure 3:**
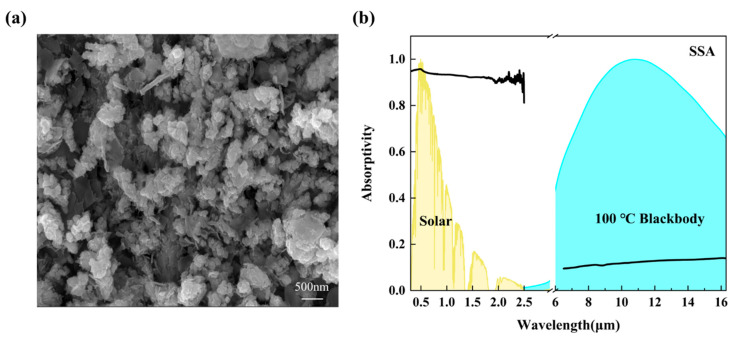
(**a**) SEM image of the selective solar absorber. (**b**) Actual spectral absorptivity of the selective solar absorber.

The three-dimensional porous copper nanostructures, formed by the solution leaching process of SSA, are crucial to its excellent optical properties. The SSA exhibits a homogeneous black appearance, with SEM characterization (Figure 3a) revealing uniformly distributed copper nanoparticles across the surface. Quantitative analysis demonstrates excellent particle dispersion with minimal agglomeration, as evidenced by the narrow size distribution. The micro-nanocomposite morphology observed in the SEM image significantly enhances the material’s specific surface area, facilitating multiple reflections and absorptions of light. The expanded absorption spectral range imparts broadband absorption properties to the SSA. This innovative structural design overcomes the optical limitations of traditional planar materials, allowing the material to capture solar radiation energy more efficiently.

Under ideal conditions, the average absorptivity of SSA peaks at 1 in the solar wavelength range (0.3–2.5 μm). In contrast, it drops to 0 in the mid-infrared region (2.5–20 μm).

The spectral analysis results of SSA (Figure 3b) demonstrate exceptional optical performance across different wavelength ranges. In the solar spectrum (0.3–2.5 μm), the material exhibits an average absorptivity of 0.91, significantly surpassing the absorption capabilities of conventional materials such as wood and ceramics [41]. In the long-wave infrared band, SSA shows a remarkably low emissivity of 0.12, indicating effective suppression of thermal radiation losses. The spectrally selective properties of the material enable the SSA to efficiently absorb solar radiation while minimizing heat loss to the environment through infrared radiation. This efficient light-to-heat conversion mechanism provides a solid energy foundation for seawater evaporation, greatly enhancing the evaporation rate and strongly supporting the efficient operation of the freshwater collection system.

### 4.2. Condensation Side Performance Evaluation

**Figure 4:**
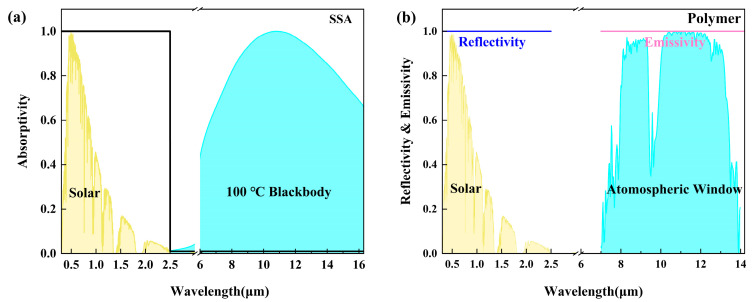
(**a**) Ideal spectral absorptivity for selective solar absorptivity. (**b**) Ideal reflectivity and emissivity of polymer coating.

**Figure 5:**
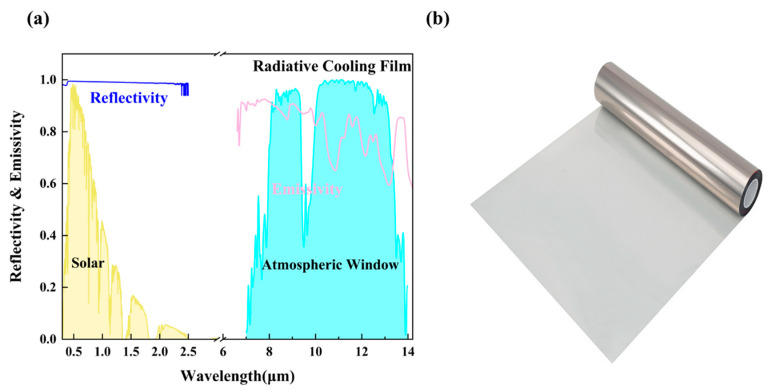
(**a**) Physical view of the radiative cooling film. (**b**) Actual solar spectral reflectivity and thermal LWIR spectral emissivity of radiative cooling film.

For optimal cooling performance, the radiative cooling film should have an average reflectivity of 1 in the solar wavelength range and an average emissivity of 1 in the long-wave infrared region (Figure 4). Spectral analysis results indicate an average reflectivity of up to 0.98 in the near-infrared (NIR) solar spectral band (0.3–2.5 μm) and an average emissivity of 0.75 in the long-wave infrared (LWIR) band. These results confirm the material’s high reflectivity in the NIR band and its efficient thermal radiation in the LWIR band (Figure 5a), both of which contribute to the formation of a stable condensation surface.

**Figure 6:**
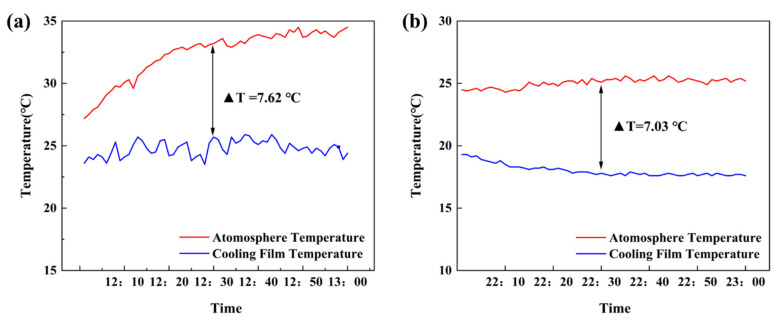
(**a**) Radiative cooling film and atmospheric temperature in the 12:00–13:00 outdoor experiment. (**b**) Radiative cooling film and atmospheric temperature in the 22:00–23:00 outdoor experiment.

Experimental measurements from the temperature monitoring system (Figure 5b) highlight the exceptional performance of the radiative cooling film under clear-sky conditions, achieving efficient sub-ambient cooling throughout the day. Notably, during peak solar irradiance at noon, when ambient temperatures reached their highest, the film achieved an average temperature reduction of 7.62 °C, with a maximum cooling of 10.4 °C (Figure 6a). This breakthrough stems from the film’s spectrally selective optical design, which integrates high solar reflectance (0.3–2.5 μm) with strong thermal emissivity (8–13 μm atmospheric window). The design facilitates net radiative heat loss to outer space, enabling sustained surface cooling even under intense solar irradiation. This daytime sub-ambient cooling capability ensures the film surface temperature stays below the ambient dew point, creating stable thermodynamic conditions for water vapor condensation. It effectively mitigates the negative impact of high daytime temperatures on the condensation process.

During the nighttime, the radiative cooling film achieved an average temperature reduction of 7.03 °C, with a maximum cooling of 7.8 °C (Figure 6b). This further reinforces its ability to operate continuously and efficiently throughout the day. As the ambient temperature decreases at night, the film continues to radiate energy into outer space, further lowering its surface temperature and promoting the formation of the condensation interface. The low-humidity conditions at night pose new challenges to the condensation process compared to daytime. However, the film continues to exhibit a significant cooling effect, demonstrating its high adaptability to varying environmental conditions. This feature enables the radiative cooling module to operate stably at day and night, ensuring continuous condensation efficiency in the freshwater harvesting system throughout the entire day, which is essential for increasing overall freshwater yield.

By integrating both daytime and nighttime experimental data, the radiative cooling film exhibits stable all-weather cooling performance, establishing a solid thermodynamic foundation for water vapor condensation. This further confirms the feasibility and high efficiency of the film as a core component in freshwater collection systems. Moreover, these data lay the groundwork for applying radiative cooling technology in water resource acquisition and provides an essential experimental basis for optimizing radiative cooling materials and expanding their potential applications.

### 4.3. Freshwater Collection Efficiency and Discussion

**Figure 7:**
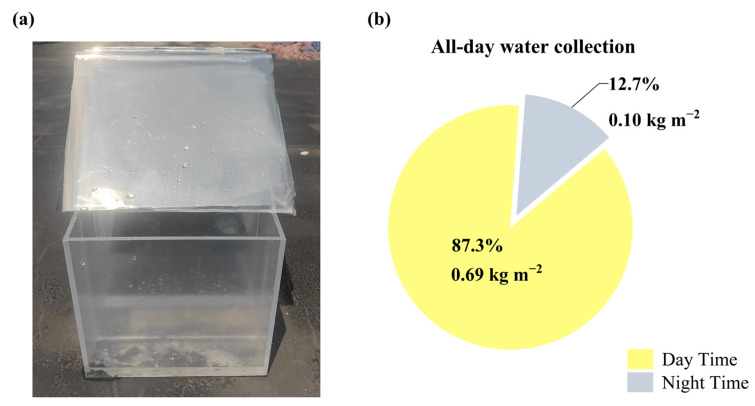
(**a**) Snapshot of the experimental setup. (**b**) Detailed data on daytime and nighttime freshwater collection on the outdoor test day.

The results of the outdoor tests are shown in Figure 7a. The experimental data demonstrate that the freshwater collection of the control group, using the conventional fixed collector, was 0.64 kg m^−2^ over 24 h. In contrast, the experimental group equipped with the solar auto-tracking device achieved a freshwater collection of 0.79 kg m^−2^, representing a 23.4% increase compared to the control group. Notably, compared with solely relying on solar thermal collection (0.42 kg m^−2^) [44], the freshwater collection capacity of this device has increased by 88%. It also demonstrates a significant growth compared to pure film condensation (0.1 kg m^−2^) [45]. Moreover, during the three-month testing period, the fluctuation range of freshwater collected by the device was maintained within ±5%, demonstrating its excellent stability.

This notable enhancement demonstrates the solar auto-tracking device’s superior performance in boosting solar thermal efficiency. By dynamically adjusting the collector angle to maintain optimal solar incidence, the device maximizes energy capture and significantly improves daytime solar-thermal conversion efficiency. This finding is consistent with existing theories on solar tracking-enhanced energy efficiency, verifying that dynamic light capture strategies play a pivotal role in freshwater harvesting systems.

Time-division data analysis showed the experimental group harvested 0.69 kg m^−2^ of freshwater during daytime (06:00–18:00), comprising 87.3% of the daily total, and 0.10 kg m^−2^ at night (18:00–06:00), accounting for 12.7% (Figure 7b). This indicates that daytime photothermal-driven evapotranspiration dominates freshwater production, while highlighting the critical role of radiative cooling technology in extending freshwater harvesting duration. At night, with decreasing ambient temperature, the radiative cooling module emits long-wave infrared radiation to outer space, reducing its surface temperature below the ambient dew point and facilitating water vapor condensation. This continuous condensation mechanism, which does not rely on traditional energy sources, ensures the sustained collection of freshwater and enables the potential for an all-weather freshwater supply.

## 5. Conclusions

In this study, an all-weather freshwater collection device was successfully designed and fabricated, integrating selective solar absorption, photoelectric sensing, and sky radiative cooling mechanisms. The SSA, fabricated using room-temperature “dip-dry” technology based on a chemical substitution reaction, shows spectral selectivity with 0.95 solar absorptivity and 0.12 infrared emissivity, enabling efficient photothermal conversion. When solar tracking is integrated into the system, freshwater production is increased by 23.4%. The radiative cooling film maintains substantial sub-ambient cooling of 7.62 °C (daytime) and 7.03 °C (nighttime), ensuring continuous vapor condensation. The experimental data showed that the average daily freshwater collection of the device was 0.79 kg m^−2^, with an energy consumption of 0.033 kg m^−2^ h^−1^ for the produced water. The freshwater collection efficiency of the device fluctuated within ±5% over three months of continuous testing, demonstrating excellent stability. The device operates solely on solar energy, effectively overcoming the high energy consumption and carbon emission limitations of traditional desalination technologies. This provides a groundbreaking solution to the global freshwater crisis.

## Data Availability

The original contributions presented in this study are included in the article and Supplementary Materials. Further inquiries can be directed to the corresponding author.

## Supplementary Materials

The following supporting information can be downloaded at: https://www.mdpi.com/article/10.3390/ma18132967/s1, Figure S1: Schematic diagram related to the solar auto-tracking system.
