# Supplementary material for: All-Day Freshwater Harvesting Using Solar Auto-Tracking Assisted Selective Solar Absorption and Radiative Cooling
Source: Materials (Basel). 2025 Jun 23;18(13):2967. doi: 10.3390/ma18132967 (PMC12251293; doi:10.3390/ma18132967)
Supplement: Supplementary file 1 [file materials-18-02967-s001.zip › materials-3697528-supplementary.pdf]

All-Day Freshwater Harvesting Using Solar Auto-Tracking Assisted Selective Solar Absorption and Radiative Cooling

Jing Luo, Haining Ji*, Runteng Luo, Xiangkai Zheng, Tianjian Xiao

School of Physics and Optoelectronics, Xiangtan University, Xiangtan, 411105, China

sdytjhn@xtu.edu.cn

To facilitate readers in having a clearer understanding and reproducing the solar tracking device, the schematic diagram, physical diagram, and circuit connection diagram of the device are presented as follows.

Figure S1(a) presents both the schematic diagram and actual photos of the system setup, offering a clear overview of its overall architecture and appearance. Figure S1(b) illustrates the wiring connections among the TDA8222A chip, photoresistors, 10 K resistors, motors, and power supply, enabling a lucid understanding of the electrical interconnections between components. (The white deposits on the selective solar absorber(SSA) in the physical image are salt residues resulting from the evaporation of seawater during the experiment.)

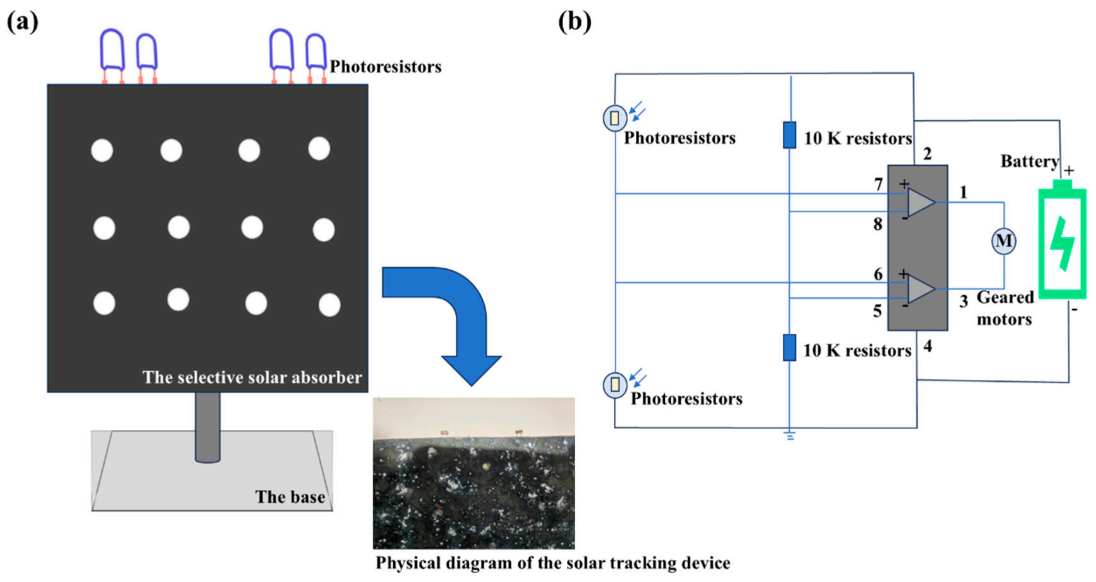

Figure S1. Schematic diagram related to the solar auto-tracking system
